# One-Step Fabrication of Porous Membrane-Based Scaffolds by Air-Water Interfacial Phase Separation: Opportunities for Engineered Tissues

**DOI:** 10.3390/membranes12050453

**Published:** 2022-04-23

**Authors:** Iris Allijn, Nikola du Preez, Małgorzata Tasior, Ruchi Bansal, Dimitrios Stamatialis

**Affiliations:** 1 Advanced Organ Bioengineering and Therapeutics, Faculty of Science and Technology, TechMed Center, University of Twente, 7500 AE Enschede, The Netherlands; nikola_dupreez@hotmail.com (N.d.P.); m.m.tasior@student.utwente.nl (M.T.); d.stamatialis@utwente.nl (D.S.); 2 Translational Liver Research, Department of Medical Cell Biophysics, Faculty of Science and Technology, TechMed Center, University of Twente, 7500 AE Enschede, The Netherlands; r.bansal@utwente.nl

**Keywords:** air-water interfacial phase separation, membrane-based scaffolds, poly (trimethylene carbonate), tissue engineering

## Abstract

Common methods for fabricating membrane-based scaffolds for tissue engineering with (hydrophobic) polymers include thermal or liquid-phase inversion, sintering, particle leaching, electrospinning and stereolithography. However, these methods have limitations, such as low resolution and pore interconnectivity and may often require the application of high temperatures and/or toxic porogens, additives or solvents. In this work, we aim to overcome some of these limitations and propose a one-step method to produce large porous membrane-based scaffolds formed by air-water interfacial phase separation using water as a pore-forming agent and casting substrate. Here, we provide proof of concept using poly (trimethylene carbonate), a flexible and biocompatible hydrophobic polymer. Membrane-based scaffolds were prepared by dropwise addition of the polymer solution to water. Upon contact, rapid solvent–non-solvent phase separation took place on the air-water interface, after which the scaffold was cured by UV irradiation. We can tune and control the morphology of these scaffolds, including pore size and porosity, by changing various parameters, including polymer concentration, solvent type and temperature. Importantly, human hepatic stellate cells cultured on these membrane-based scaffolds remained viable and showed no signs of pro-inflammatory stress. These results indicate that the proposed air-water interfacial phase separation represents a versatile method for creating porous membrane-based scaffolds for tissue engineering applications.

## 1. Introduction

Porous polymeric scaffolds have broad applications in drug delivery, microfluidics, filtration and tissue engineering (TE) [1]. As for filtration, polymeric membrane-based scaffolds for TE require interconnected pores that are large enough to accommodate cell infiltration, nutrient supply and ingrowth of blood vessels while maintaining pore integrity and mechanical properties to mimic tissue-specific characteristics [2].

To fabricate synthetic porous polymeric scaffolds from hydrophobic polymers, methods such as phase inversion, sintering [3], electrospinning [4,5], particle leaching [6,7], stereolithography [7,8], phase separation micro molding [9], freeze drying [10] and gas foaming [7,11] are used. Furthermore, a combination of techniques is often needed to achieve the desired scaffold properties. For example, to create hierarchical pores, a combination of particle leaching and stereolithography can be applied [12]. However, membrane-based scaffolds produced by such methods have limitations for TE, such as poor control over pore size, limited porous hierarchy, undesirable morphology that does not mimic tissues or limited construct volume. Besides synthetic hydrophobic polymers, natural hydrogels (e.g., gelatin and alginate) made from hydrophilic polymers are often used for TE applications; however, these alternatives also present processing and handling challenges.

In this work, we propose a one-step method to fabricate large porous polymeric membrane-based scaffolds based on air-water interfacial phase separation, using water as a pore-forming agent and casting substrate. Here, a hydrophobic polymer was dissolved in a suitable non-volatile, biocompatible solvent and was subsequently slowly added dropwise onto the surface of ultrapure water. Phase separation occurred on the air-water interface, and the floating porous polymeric layer was subsequently crosslinked via UV irradiation. For proof of concept, we applied this method to the preparation of poly(*trimethylene carbonate*) (PTMC) scaffolds. PTMC is amorphous, flexible and biodegradable through surface erosion without acidic degradation products [13].

We investigated various fabrication parameters, such as the time on water before photo crosslinking, solvent and temperature to control the pore size and porosity of the scaffolds. Finally, we performed preliminary in vitro studies using human hepatic stellate (LX2) cells and assessed their suitability for TE applications.

## 2. Materials and Methods

### 2.1. Materials

Tin(II)2-ethylhexanoate (Sn(Oct)_2_), trimethylamine (TEA), methacrylic anhydride (MAAh), deuterated chloroform, 1,6-hexanediol, 2-hydroxy-4-(2-hydroxyethoxy)-2-methylpropiophenone (Irgacure 2959), hydroquinone (HQ), tris(hydroxymethyl)aminomethane (TRIS), resazurin sodium salt, gelatin from porcine skin (90–110 g, type A), GenElute Mammalian Total RNA Miniprep Kit, L-Glutamine, sodium dodecyl sulfate polyacrylamide (SDS), Tris-buffered saline with Tween-20 (TBST-20), trypsin-ethylenediaminetetraacetic acid (EDTA), polyvinylidene fluoride (PVDF) Western blotting membranes and human primers for genes IL-1β, IL-6, iNOS and GAPDH were purchased from Sigma-Aldrich (Zwijndrecht, The Netherlands). Propylene carbonate (PC), ethanol (EtOH) absolute, N-methyl-2-pyrrolidone (NMP) and calcium hydride were obtained from Merck Millipore (Darmstadt, Germany). Methanol, dichloromethane (DCM), DMSO, diethyl ether, dimethylformamide (DMF) and bovine serum albumin (BSA) were purchased from VWR (Amsterdam, The Netherlands). Trimethylene carbonate (TMC) was obtained from Huizhou Foryou Medical Devices Co (Huizhou, China). High-vacuum grease was purchased from Dow Corning (Midland, MI, USA). Fetal bovine serum (FBS) was obtained from Lonza (Verviers, Belgium). Dulbecco’s modified Eagle’s medium (DMEM) with GlutaMAX, phosphate-buffered saline (PBS), penicillin-streptomycin (pen-strep), RPMI-1640 medium, Pierce Western blot transfer buffer (10X), Pierce ECL Plus Western blotting substrate (enhancer solution), SuperSignal, PageRuler (10-250 kDa) and Western blotting filter paper were acquired from Fisher Scientific (Naarden, The Netherlands). Phorbol-12-myistate-13-acetate (PMA) was purchased from Cayman Chemicals (Ann Arbor, MI, USA), Tween-20 from Acros Organics (Antwerp, Belgium) and Novex WedgeWell 4–20% Tris-glycine gel from Invitrogen (Carlsbad, CA, USA). SYBR Green Supermix and iScript cDNA Synthesis Kit were purchased from Bio-Rad. Anti-type I collagen (goat) was obtained from Southern Biotech (Birmingham, AL, USA), and rabbit anti-goat and goat anti-rabbit polyclonal antibodies were obtained from Dako (Glostrup, Denmark).

### 2.2. Ring-Opening Polymerization of TMC and Functionalization of PTMC

Linear two-armed PTMC was synthesized by ring-opening polymerization (ROP) of TMC as described previously [14]. Briefly, polymerization was conducted in an argon atmosphere at 130 °C for three days with Sn (Oct)_2_ as a catalyst. Hexanediol (0.1 mmol/g monomer to obtain an oligomer with Mn ≈ 10 kg/mol) was used as an initiator for PTMC. Cooled-down oligomers were functionalized in DCM (dried over calcium hydride and distilled) by reaction with MAAh (6 mol/mol initiator) in the presence of TEA (6 mol/mol initiator) and HQ (0.1% *w*/*w* to monomer) for five days at RT (Figure S1). Subsequently, functionalized PTMC (PTMC-dMA) was purified by precipitation in ice-cold methanol. The obtained macromers were dried in the dark at RT overnight and in vacuo for one week until constant weight was achieved. The (non-)functionalized macromers were dissolved in deuterated chloroform to determine the TMC conversion, the number average molecular mass (Mn) and the degree of functionalization (df) by ^1^H-NMR (Bruker Ascend 400/Avance III 400 MHz NMR spectrometer, Bruker, Billerica, MA, USA).

### 2.3. Air-Water Interfacial Phase Separation for PTMC-dMA Scaffold Fabrication

PTMC-dMA was dissolved in DMSO (15–30% *w*/*w* of total solution) with Irgacure 2959 (3% *w*/*w* of polymer) as a photoinitiator under gentle stirring in the dark until dissolved. To create the polymeric networks, milliQ water was added to a Petri dish (Ø = 3 cm for small and Ø = 9 cm for large scaffolds). Subsequently, the PTMC-dMA solution was carefully deposited dropwise on the water surface, creating a polymer layer floating on the water. To create a large scaffold, additional polymer solution drops were added to the scaffold until it became opaque.

PTMC-dMA in DMSO (25% *w*/*w*) was allowed to spread on the water surface for 1, 10, 30, 60, 180, 300 and 420 min before photo crosslinking on the water for 2 min. Photo crosslinking was performed at 365 nm at 36 W (4 lamps × 4 Watt) with an intensity of ~6 mW/cm^2^ (Geeek 36 W UV Gel lamp, Dermarolling, Holten, The Netherlands) at a distance of 3 cm from the lamps. The scaffolds were collected from the water, swollen in PC and slowly extracted using EtOH to remove the sol fraction. For further analysis, scaffolds with time on water of 10, 60 and 180 min were selected based on their morphological differences and named Sc1, Sc2 and Sc3, respectively. To tailor the pore morphology, an N_2_ flow was applied for 10 and 60 min on the top of the scaffold during phase separation and prior to photo crosslinking. Besides DMSO, we also applied our method using PTMC-dMA dissolved in NMP, DMF, PC or chloroform (25% *w*/*w*) to investigate the influence of the solvent properties on scaffold formation and morphology after 10 min. Table S1 describes all investigated conditions.

### 2.4. Characterization of PTMC-dMA Scaffolds

The gel content of the scaffolds was determined by comparing the dry mass of the scaffold after extraction (m_1_) with the solvent-corrected polymer mass before photo crosslinking (m_corr_) using the following equation:

Gel content = (m_1_/m_cor_) × 100%, (1)

The scaffold porosity was determined by comparing the dry mass (m_1_) of the PTMC-dMA scaffolds to the theoretical weight of solid PTMC (density ρ = 1.31 g/cm^3^) of the same volume (V) [15] using the following equation:

Porosity = {1 − (m_1_/ρ V)}, (2)

Scanning electron microscopy (SEM) was used to assess scaffold morphology. Scaffolds were gold-coated for 60 s (Cressington Sputter Coater 108Auto, Cressington, Watford, England) and imaged using a JEOL JSM-IT1000 SEM (Jeol Europe, Nieuw-Vennep, The Netherlands). The surface pore areas were measured in a 926 × 926 μm (0.86 mm^2^) grid on 30x magnified scanning electron micrograph using Image J 1.52p (NIH, Bethesda, MD, USA).

To demonstrate the open cell structure and interconnectivity of the pores, a clean water permeability test was performed as described in [16], with some modifications. Briefly, the scaffolds were cut in circles with a surface area (*A*) of 4.9 cm^2^ and mounted in a 3 mL dead-end Amicon cell (Pmax 5.2 bar, Merck Millipore, Darmstadt, Germany) with the air side facing the feed solution. The cell was subsequently connected to a pressurized tank with milliQ water (Purelab Flex, Elga, Salmenkipp, Breukelen, The Netherlands). Prior to measurement, the scaffolds were wetted under low pressure for 30 min. Afterwards, the clean water flux (flux (*J*) = *V*/(*At*)) was determined by measuring the water volume (*V* in *L*) that came through the membranes at different transmembrane pressures (ΔP in *bar*) for 20 min (*t* in *h*), starting with the same pressure as that used for wetting. The permeated water through Sc1 was determined at a TMP of 1.5, 2 and 2.5 bar and through Sc2 and Sc3 at 0.5, 0.75 and 1 bar. The measurement ended at the starting pressure to control for compaction behavior of the membrane. The water permeance, P, was calculated from the slope of the linear part of the flux versus ΔP curve:

J = P × ΔP, (3)

The mechanical properties of the photo-crosslinked networks were assessed in the dry state. For this, at least three replicates (100 mm × 5 mm × 0.5 mm) per scaffold were measured in a tensile tester (Zwick Z020, Zwick/Roell, Ulm, Germany) with a 500 N load cell, an initial grip-to-grip separation of 30 mm and a test speed of 50 mm/min. The Young’s modulus at 1–4% elongation, maximal stress (kPa) and elongation at break (%) were determined. The toughness (N/mm^2^) of the samples was defined as the area under the stress–strain curve.

### 2.5. Human Hepatic Stellate (LX2) Cell Culture on PTMC-dMA Scaffolds

Hepatic stellate (LX2) cells were maintained in growth medium consisting of DMEM with GlutaMAX, 10% (*v*/*v*) FBS and 1% (*v*/*v*) Pen/Strep at 37 °C in a humified incubator containing 5% CO_2_. The cells were passaged every four days, when confluent, by washing with PBS and using 0.25% (*v*/*v*) trypsin-EDTA (0.25% trypsin, 0.02% EDTA in Hank’s Balanced Salt Solution). Prior to seeding for cell experiments, the cells were washed twice with PBS, dissociated using 0.25% (*v*/*v*) trypsin-EDTA and counted with a hemocytometer. The cells were seeded on both the air side and water side of the scaffolds at a 100,000 cells/mL concentration. After 24 h, the LX2-conditioned medium was collected and stored at −80 °C for further use. The metabolic activity of the LX2 cells was determined using Alamar blue solution (440 µM resazurin sodium salt in PBS). Medium containing 10% *v*/*v* Alamar blue was added to the cells, and after 4 h of incubation at 37 °C in a humified incubator containing 5% CO_2_, fluorescence was measured at an excitation/emission of 560/590 nm using a spectrophotometer (Victor3 Wallac, PerkinElmer, Groningen, The Netherlands). The experiment was repeated three times (n = 3).

### 2.6. Protein Analysis of the LX2-Conditioned Medium

To separate the proteins with SDS-PAGE, 150 µL of the LX2-conditioned medium was freeze-dried (Scala Scientific Freeze Dryer, Scala-Scientific, Ede, The Netherlands). Subsequently, the samples were resuspended in 20 µL MilliQ and heated for 5 min at 95 °C in a dry bath. The prepared samples were loaded on 10% Tris-glycine gels (Life Technologies, Carlsbad, CA, USA), followed by transfer to a PVDF membrane (Roche) using a standardized protocol. After blotting, the PVDF membranes were blocked for 1 h in blocking buffer (5% BSA containing 1x TBST (Tris-buffered saline Tween, Life Technologies)). PVDF membranes were incubated with collagen I antibody (goat polyclonal, 1:300) prepared in blocking buffer and incubated overnight at 4 °C. Afterwards, the membranes were washed three times in 1x TBST for 5 min and incubated with specific secondary antibodies (HRP-conjugated rabbit anti-goat 1:100 and HRP-conjugated goat anti-rabbit) prepared in blocking buffer and incubated for 1 h. Finally, the membranes were washed three times in 1x TBST for 5 min and incubated for ca. 5 min with freshly prepared enhancer solution as per the manufacturer’s instructions. The excess was tapped off, and the membranes were placed on a chemiluminescence plate and exposed in a ProteinSimple FluorChem M imaging system to obtain images of the protein bands.

### 2.7. Gene Analysis of Human THP-1 Monocytes Cultured in LX2-Conditioned Medium

Human THP-1 monocytic cell line (obtained from ATCC) was maintained in growth medium consisting of RPMI-1640 with 10% (*v*/*v*) FBS, 1% (*v*/*v*) Pen/Strep and 2 mM glutamine at 37 °C in a humified incubator containing 5% CO_2_. To differentiate the monocytes from (M0) macrophages, the cells were seeded in a 24-well plate at 500,000 cells/mL and incubated in growth medium with 0.1% (*v*/*v*) of the PMA stock solution (100 μg/mL in PBS) for 16–18 h at 37 °C in a humified incubator containing 5% CO_2_. The differentiation of the THP-1 monocytes to (M0) macrophages could be determined by examining the cell adherence to the wells plate.

To examine the effect of the LX2-conditioned medium, the (M0) macrophages were incubated with 50% *v*/*v* conditioned medium for 6–8 h at 37 °C in a humified incubator containing 5% CO_2_. Cells were then lysed using RNA lysis buffer, and total RNA was isolated using a GenElute Total RNA Miniprep kit (Sigma) according to the manufacturer’s instructions. The RNA concentration was quantified using a NanoDrop^®^ ND-1000 spectrophotometer (Thermo Scientific, Waltham, MA, USA). Total RNA (1 µg) was reverse-transcribed using an iScript cDNA synthesis kit (Bio-Rad, Hercules, CA, USA). Real-time PCR was performed using 20 ng of cDNA, pretested gene-specific primer sets (IL-1β, IL-6, iNOS and GAPDH (housekeeping)) and a 2x SensiMix SYBR and fluorescein kit (Bioline GmbH, QT615-05, Luckenwalde, Germany) according to the manufacturer’s instructions. Finally, cycle threshold (Ct) values were normalized to the reference gene 18 s rRNA, and relative expressions were calculated using the 2-ΔΔCt method.

### 2.8. Statistical Analysis

All the results are expressed as the mean ± standard deviation (SD). All graphs were made and all statistical analyses were performed using GraphPad Prism (GraphPad Prism, La Jolla, CA, USA). Statistical analysis of characterization data was performed using a one-way ANOVA with a Tukey’s multiple comparison post-test. Differences were considered significant when * *p* ≤ 0.05, ** *p* ≤ 0.01 or *** *p* ≤ 0.001 and non-significant (ns) when *p* > 0.05.

## 3. Results and Discussion

### 3.1. Air-Water Interfacial Phase Separation for Porous Membrane-Based Scaffold Formation

**Figure 1:**
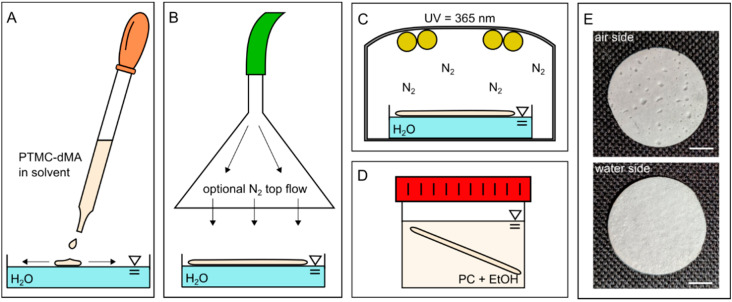
One-step porous membrane-based scaffold formation by air-water interfacial phase separation. (**A**) PTMC-dMA in a solvent is added dropwise to water. (**B**) The PTMC-dMA spreads on the surface of the water over time. An optional N_2_ top flow (see arrows) can be applied to enhance the pore gradient. (**C**) The PTMC-dMA floating layer is photo-crosslinked while on the water. (**D**) The scaffold is taken from the water, and the sol fraction is removed by swelling in propylene carbonate (PC), followed by extraction with EtOH. (**E**) Photographs of the air side (top) and water side (bottom) of the scaffold. Scale bars are 1 cm.

When a solvent with a low surface tension is dropped onto a high-surface-tension solvent or solution (e.g., water), spontaneous spreading occurs—the so-called Marangoni flow. Spreading is dictated by the (positive) spreading coefficient, S, which is defined by the surface tensions of the liquid droplet and liquid substrates [17]. When PTMC-dMA (dissolved in DMSO, which has a lower surface tension than water) is carefully dropped on water, spontaneous spreading occurs because S > 0, and a floating polymeric layer is formed (Figure 1, Video S1). However, when the non-solvent EtOH is used as a substrate, which has a lower surface tension than DMSO, the spreading coefficient is S < 0, so the PTMC-dMA solution balls on the water and sinks. Because water and DMSO are freely miscible (Table 1), solvent exchange occurs immediately, and PTMC-dMA rapidly desolvates on the air-water interface and forms a floating precipitate. This precipitate layer can subsequently be fed with additional PTMC-dMA solution drops to grow until solvent exchange is complete. The solvent exchange of water and DMSO results in the formation of water pockets in the polymeric network, which contribute the porous character and therefore play an important role in the formation of the membrane-based scaffold. This polymeric layer is subsequently photo-crosslinked to obtain a stable porous structure with a clear morphological difference on the air and water side, as well as in terms of shape memory (Video S2).

### 3.2. Essential Parameters for One-Step PTMC-dMA Porous Scaffold Fabrication on the Air-Water Interface

First, we successfully synthesized PTMC by ring-opening polymerization (ROP) from TMC functionalized with two methacrylate groups (PTMC-dMA) (Figure S1). The TMC conversion was 96% with the Mn 8.6 kg/mol, and 85% functionalization was achieved, as determined by ^1^H-NMR. All porous scaffolds in this study were prepared with the same batch of PTMC-dMA. Before preparing the scaffolds for in vitro biocompatibility studies, we extensively examined the essential parameters—polymer concentration, solvents and temperature—to determine the optimal settings for one-step porous scaffold formation, as summarized in Table S1.

#### 3.2.1. PTMC-dMA Concentration Determines Homogeneity of the Porous Scaffolds

**Figure 2:**
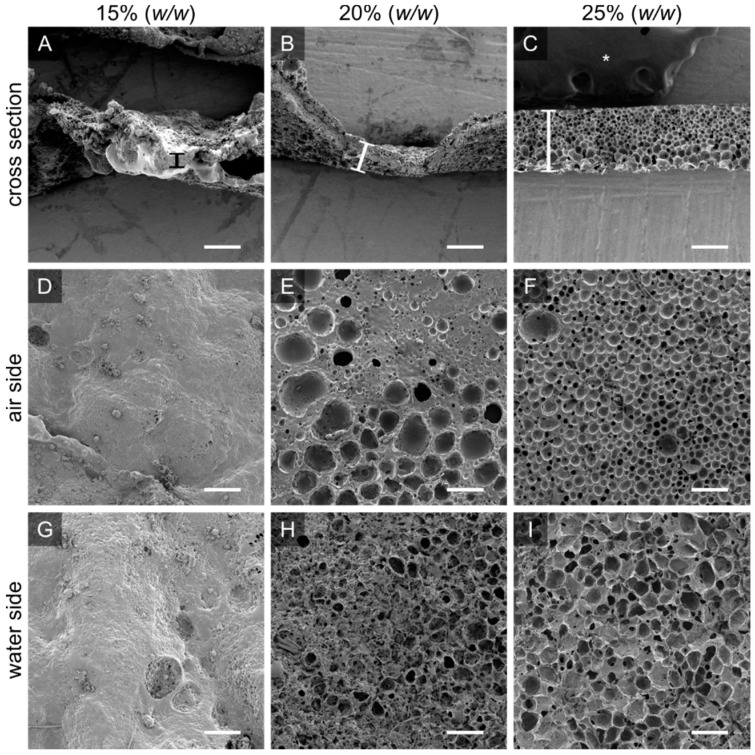
The initial PTMC-dMA concentration is crucial for homogenous porous scaffold formation. Scanning electron micrographs are shown for PTMC-dMA scaffolds prepared at room temperature for ten minutes with three PTMC-dMA concentrations. The top panels (**A**–**C**) show the cross sections with the air side facing upwards. The thickness is indicated by a vertical bar. The middle panels (**D**–**F**) show the air side, and the bottom panels (**G**–**I**) show the water side of the scaffolds. Scale bars are 200 µm. The asterisk (*) indicates the carbon tape used to stick the samples to the SEM stub.

In order to create homogenous scaffolds with a consistent thickness and porosity, the suitable concentration of the initial polymer solution was determined. A PTMC-dMA concentration of 15% (*w*/*w* of total) resulted in a polymer solution that flowed easily on the water. The DMSO in the polymer solution is more accessible for the water; therefore, fast solvent–non-solvent exchange occurred. The PTMC-dMA, in turn, rapidly desolvated, resulting in a disorganized and heterogeneous polymer network. A concentration of 20% (*w*/*w* of total) resulted in pores on both the air and the water side, with a denser middle. A PTMC-dMA concentration of 25% (*w*/*w* of total) resulted in a thick and fairly homogenous porous scaffold from air to the water surface (Figure 2). The PTMC-dMA with a concentration >25% (*w*/*w* of total), on the other hand, was close to the solubility limit and too viscous to apply. Therefore, to create a homogenous porous scaffold, a PTMC-dMA concentration of 25% (*w*/*w* of total) was used in the following experiments.

#### 3.2.2. Solvents Influence Pore Morphology and Scaffold Thickness

**Figure 3:**
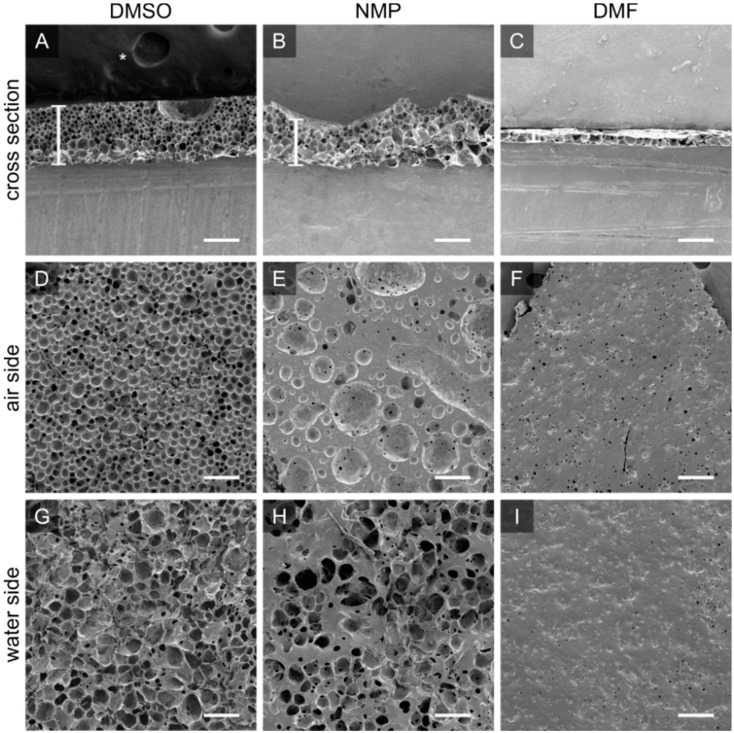
PTMC-dMA dissolved in DMSO, NMP and DMF results in different porous scaffolds. Scanning electron micrographs for PTMC-dMA scaffolds with an initial concentration of 25% (*w*/*w*) in DMSO (scaffold Sc1), NMP and DMF prepared by air-water interfacial phase separation for 10 min. The top panels (**A**–**C**) show the cross sections of the scaffolds, with the air side facing upwards. Scaffold thickness is indicated by a white vertical bar. The middle panels (**D**–**F**) show the air side, and the bottom panels (**G**–**I**) show the water side of the scaffolds. Scale bar is 200 µm. The asterisk (*) indicates the carbon tape that was used to stick the samples to the SEM stub.

The PTMC-dMA solvent influences scaffold formation on the air-water interface due to the changing solvent properties and miscibility with water (Figure 3). PTMC-dMA dissolved in NMP spread in a similar way on the water to PTMC-dMA in DMSO, where the spreading coefficient, S > 0; however the resulting scaffolds displayed large craters on the air side (Figure 3B). When dissolved in DMF, the spreading coefficient, S, was higher than for DMSO and NMP, and therefore the polymer solution spread rapidly, and a thin scaffold was formed with a porous inside but without pores on the outside (Figure 3). When dissolved in PC, no scaffold was formed on the water surface due to the poor water miscibility and non-volatile nature of PC. Because of this combination, PTMC-dMA did not spread well on the water surface, and no scaffold was formed. When a volatile solvent, such as chloroform, was used, the spreading coefficient, S, increased, and due to the very poor miscibility with water, hardly any solvent–non-solvent exchange occurred. However, the chloroform evaporates rapidly, resulting in very thin non-porous films similar to those described previously in the literature [17,18,19,20,21,22,23,24,25]. In summary, a solvent with S > 0, is freely miscible with water and does not evaporate results in the formation of a porous scaffold. In this paper, we focused on porous scaffold formation of PTMC-dMA dissolved in DMSO because of the homogenous pore formation and the relative biocompatibility of DMSO [26], which is important for TE applications.

#### 3.2.3. Temperature Affects PTMC-dMA Spreading on Water

Temperature also plays a crucial role in tuning the properties of the porous PTMC-dMA membrane-based scaffolds. At room temperature (RT), the PTMC-dMA precipitate on the water continues to reorganize due to the amorphous nature of PTMC and its low glass transition temperature (Tg) of around −16–+7.6 °C [27]. Furthermore, the temperature of the water phase influences the kinetics of the system; the surface tension of water decreases with increased temperature. Meanwhile, an increased temperature of 40 °C makes the PTMC-dMA solution because of the increased distance from both the freezing point of DMSO and the Tg of PTMC. This results in a faster expansion and precipitation of the PTMC-dMA on the water surface, tearing the formed floating porous layer apart over time. This phenomenon is in line with previously reported thin, non-porous floating films on water [22]. Cold water with a temperature of 4 °C causes the PTMC-dMA solution to immediately sink, resulting in a clump similar to that achieved when the PTMC solution was dropped on water with a reduced surface tension caused by a detergent (Figure S2).

Therefore, the concentration of the PTMC-dMA solution, the temperature of the water and the solvent are important parameters in the robust and controllable fabrication of porous scaffolds. In this case, a 25% *w*/*w* PTMC-dMA solution in DMSO on RT water successfully resulted in large porous scaffold (>cm in diameter) suitable for TE applications. All further experiments were performed using these conditions. To the best of our knowledge, this is the first time that air-water interfacial phase separation has been used to produce large porous membrane-based scaffolds with controllable pore size and porosity in a single step.

### 3.3. Scaffold Porosity and Pore Size Can Be Controlled without the Addition of Porogens

**Figure 4:**
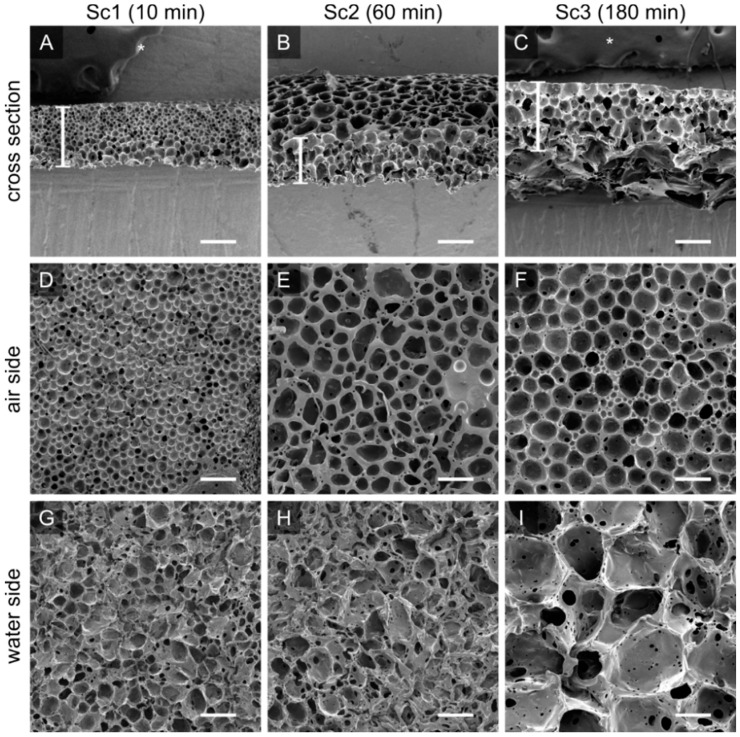
The porosity and pore size of the PTMC-dMA scaffolds is controlled by the floating time on the water before photo crosslinking. Scanning electron micrographs are shown for PTMC-dMA scaffolds Sc1, Sc2 and Sc3 with an initial concentration of 25% (*w*/*w*) and a time on water of 10 min (**A**,**D**,**G**), 60 min (**B**,**E**,**H**) and 180 min (**C**,**F**,**I**). Scale bars are 200 µm. The asterisk (*) indicates the carbon tape that was used to stick the samples to the SEM stub.

To create a robust and porous membrane-based scaffold, we used PTMC-dMA (25% *w*/*w*) dissolved in DMSO added dropwise to purified water at RT. As the PTMC-dMA in DMSO was slowly dropped, it precipitated on the water surface due to the non-solvent-induced phase separation and the positive spreading coefficient. However, because PTMC-dMA is an amorphous polymer with low Tg [27], the floating polymer layer was still flexible and rearranged over time at RT until the network was crosslinked with UV irradiation. By varying the length of time the PTMC-dMA layer is on the water before photo crosslinking (‘time on water’), the surface pore size and porosity can be controlled (Figure S3). Even hierarchical porous structures could be created without additional porogens (Figure 4F). In this manner, three different porous scaffolds with respective time on water of 10, 60 and 180 min were selected as specified in brackets () and referred to as Sc1, Sc2 and Sc3, respectively (Figure 4, Table 2). These scaffolds displayed porosities of 70, 75 and 81%, respectively, whereas the total number of pores on both the air and the water side decreased. This is supported by the increasing pore diameter of 21, 44 and 52 μm on the air side and 74, 98 and 295 μm on the water side for Sc1, Sc2 and Sc3, respectively (Figure 4 and Table 2). These pores are generally large enough for human cells to reside in.

### 3.4. Mechanical Properties

To determine the mechanical properties of the porous membrane-based scaffolds, tensile strength tests were performed. Although the pore sizes and porosities changed significantly, the Young’s modulus, dL at break, maximum stress and toughness were not significantly different. Compared to a non-porous PTMC film [14], the Young’s modulus decreased about four times for scaffolds with a porosity of >70% (Table 2).

The porosity and morphology of a porous PTMC-dMA membrane-based scaffold can be controlled without affecting the mechanical properties too much; however, a decreasing trend is visible towards the more porous scaffold Sc3. This trend is similar to what was described previously in thin porous PTMC membranes [9]. The Young’s modulus decreases with increased porosity; however, the extent of this decrease is dependent on the shape of the pores [11].

### 3.5. Pore Structure of PTMC-dMA Membrane-Based Scaffolds

The water permeance of the membrane-based scaffolds was estimated from the clean water flux experiments, which confirmed an open cell structure for Sc2 and Sc3, as observed by scanning electron microscopy (SEM) (Figure 4 and Table 2). For the Sc1 scaffold, the water permeance was much lower, which might have been caused by high tortuosity or closed pores. The porous PTMC-dMA scaffolds, when cast under RT air conditions (Figure 4), were asymmetric, with larger pores on the water side and smaller pores on the air side. When casting the scaffolds, small droplets of non-solvent popped out on the air side, which ultimately formed surface pores. When using a short time on water (e.g., Sc1) there was no time to form open cell structures, and larger craters were observed on the air side of the scaffolds. This resulted in very low pore connectivity.

To enhance the pore size gradient and avoid the formation of large craters, an N_2_ flow above the water surface was applied (Figure 1B). This resulted in a significant reduction in pore diameter on the water side after 10 min, whereas after 60 min, the air side became quite dense, with no visible pores; the pore morphology on the water side of the membrane-based scaffolds was affected only to a limited extent (Figure S4). This might have been caused by the fact that the non-solvent (water) that popped out on the air side was flushed away by the nitrogen stream and could not form droplets to form the (larger) pores. These scaffolds, which could be interesting as TE constructs and are closed off at one side to protect cells from outside influences, are not discussed further in this paper.

### 3.6. Porous PTMC-dMA Membrane-Based Scaffolds for Liver Tissue Engineering

The highly porous PTMC-dMA membrane-based scaffolds display potentially beneficial features for TE applications. The interconnected pores allow cells to migrate into the scaffold, whereas the hierarchical pores in turn enable nutrient transport throughout the scaffold. The fabricated scaffolds were flexible and recovered their shape after deformation (Video S2). The pore size of the air side of the scaffolds was large enough for smaller human cells (i.e., hepatocytes; 25 to 30 μm), and the pores on the water side were large enough for larger human cells (i.e., adipocytes; <20 to 300 μm) to reside in [28]. The general scaffold pore morphology looked similar to the porous morphology of the extracellular matrix (ECM) of several organs, including heart [29], kidney [30] and liver [31,32]. Furthermore, in previous studies, it has been shown that cells adhere well to PTMC surfaces [33,34]. Moreover, PTMC-dMA is a promising material for clinical translation, as it degrades in vivo without toxic acidic degradation products [13]. To demonstrate in vitro biocompatibility and TE potential of these porous PTMC-dMA membrane-based scaffolds, we used the liver as our model tissue. To this end, human hepatic stellate cells (LX2) were cultured on top of both the air and the water side of the scaffolds (Figure S5) to determine metabolic activity, immune response and ECM deposition. Tissue culture polystyrene (TCPS) was used as a positive control.

#### 3.6.1. Porous PTMC-dMA Membrane-Based Scaffolds Are Biocompatible

**Figure 5:**
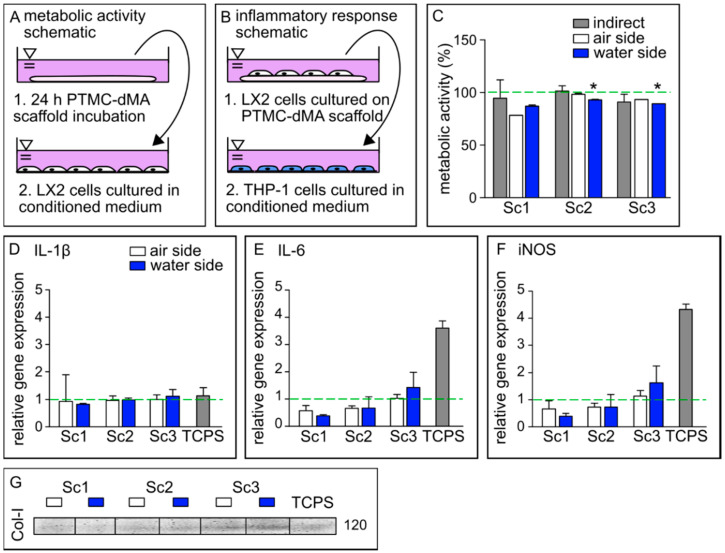
PTMC-dMA scaffolds are biocompatible and induce extracellular matrix formation. Schematics of conditioned medium used in (**A**) metabolic activity assays and (**B**) inflammatory response assays. (**C**) Metabolic activity of LX2 cells under various culture conditions after 24 h. The dashed green line indicates 100% metabolic activity of the control cells cultured on TCPS without conditioned medium. * *p* < 0.05 versus the TCPS control. Relative gene expression of (**D**) IL-1β, (**E**) IL-6 and (**F**) iNOS in M0 macrophages cultured in conditioned medium. The dashed green line indicates the relative gene expression found in M0 macrophages cultured in normal medium as a control. (**G**) Western blot of collagen 1 (Col-I) production of LX2 cells under various conditions. TCPS is the control of LX2 cells cultured on TCPS without conditioned medium.

Cell viability was measured through metabolic activity in a direct and an indirect manner. To determine possible cytotoxicity, the membrane-based scaffolds were incubated in medium for 24 h, after which the conditioned medium was transferred to LX2 cells cultured on normal TCPS (Figure 5A). After 24 h, the metabolic activity of LX2 cells was measured using an Alamar blue assay. The indirect viability of the cells was high, indicating that the PTMC-dMA itself did not leach any cytotoxic components. The cells, cultured on both sides of membrane-based scaffolds Sc1, Sc2 and Sc3 proliferated well, and the viability of the pore morphology was reduced slightly but stayed above a reasonable ~80% (Figure 5C). The smaller pores of the Sc1 scaffold had the lowest cell viability, which might have been caused by the fact that the pores are smaller than the cells; hence, the cells have to bridge the gaps. Furthermore, Sc1 showed a very low water permeability, resulting lower cell viability, which might also be caused by reduced nutrient supply due to poor pore connectivity. However, as reported previously [35], we expect that this slightly reduced viability will improve over time, as LX2 cells need to adjust to the substrate/microenvironment. Moreover, the morphological difference of the scaffolds did not influence the LX2 viability significantly; however, interconnected pores are required for 3D tissue engineering purposes, and therefore, scaffold Sc1 is not suitable.

#### 3.6.2. Porous PTMC-dMA Membrane-Based Scaffolds Do Not Induce Inflammatory Response

To investigate whether the porous membrane-based scaffolds induce an inflammatory response, the medium of LX2 cells cultured on the scaffolds was collected and used to culture human monocytes (THP-1) on TCPS. The conditioned medium of LX2 cells cultured on TCPS was used as a control. Gene expression relative to THP-1 cells cultured in normal growth medium was determined for IL-1β, IL-6 and iNOS, known to play an important role in inflammation [36] (Figure 5B,D–F).

The relative gene expression of IL-1β induced by the conditioned medium of all membrane-based scaffolds on both sides was similar to that of the TCPS control, whereas for IL-6 and iNOS, the expression was much lower. Furthermore, a trend of increased gene expression of IL-6 and iNOS could be observed in scaffolds with a larger pore size (Sc3), although still much lower than the expression induced by the control TCPS. A similar effect was observed for peripheral mononuclear cells cultured on both GelMA and TCPS, where the cytokine expression after lipopolysaccharide (LPS) stimulation was increased on TCPS compared to GelMA [37]. These results suggest that these porous PTMC-dMA membrane-based scaffolds do not propagate stellate cell activation, which is a major factor in the progression of liver fibrosis [32]. These data show that the porous PTMC-dMA Sc3 scaffolds can support cell culture for liver TE applications without inducing an inflammatory response.

#### 3.6.3. Porous PTMC-dMA Membrane-Based Scaffolds Induce Extracellular Matrix Formation by Human Stellate Cells

The Western blot results show that the LX2 cells cultured on the membrane-based scaffolds produced more collagen-I (Col-I) as an ECM marker, indicating the functional viability of these cells (Figure 5G). In particular, scaffold Sc3 displayed an increased production of Col-I when compared to the TCPS control. This could have been caused by the fact that Sc3 has larger pores that the stellate cells can easily migrate into, as well as a reduced Young’s modulus, mimicking a more natural cell environment than that of Sc1 and Sc2. Furthermore, this increased porosity could play a role, in addition to nutrients, in better reaching the cell. These results are in line with the inflammatory gene expression profile, which indicates that decreased collagen secretion reduced immune modulatory effects with Sc1 and Sc2 compared to Sc3. Therefore, because of their good metabolic activity, excellent ECM formation stimulation and low anti-inflammatory response compared to control TCPS, the porous PTMC-dMA membrane-based scaffold Sc3 is a good candidate for engineered liver tissue applications.

## 4. Conclusions and Outlook

In this paper, we presented a versatile one-step method based on air-water interfacial phase separation to fabricate flexible, highly porous PTMC membrane-based scaffolds. To the best of our knowledge, this is the first time that this method has been used for the fabrication of large porous membrane-based scaffolds using water not only as a casting substrate but also as a pore-forming agent. We tuned various membrane-forming parameters, such as polymer concentration, solvent type and floating time on the water phase, and we demonstrated that we could control well their porosity, pore gradient and thickness. The most optimal membrane-based scaffold formation (Sc1-3) was achieved with 25% *w*/*w* PTMC-dMA in DMSO and a time on water before photo-crosslinking between 10 and 180 min. Sc2 and Sc3 possessed desired structural properties for TE, such as high porosity and pore interconnectivity and were able to support human stellate (LX2) cell culture. Moreover, this fabrication method is applicable for other hydrophobic polymers. Like the parameters tailored here, one could also consider tailoring the composition of the water phase by addition of hydrophilic methacrylate-functionalized polymers, such as PEG-dMA and GelMA, which could potentially allow for fabrication of membrane-based scaffolds with an immunoprotective layer or a soft cell-adherent layer, respectively, which should be tested with other cell types to ensure its (long-term) cytocompatibility. The proposed one-step fabrication process could offer possibilities for preparing membrane-based scaffolds for TE. To achieve even larger scaffolds potentially suitable for application in large animal and/or human studies, development of an automated and controlled method of polymer drop deposition on the water surface is required based on principles of 3D printing and/or additive manufacturing.

## Figures and Tables

**Table 1:** Solvent characteristics. Water and ethanol (EtOH) are non-solvents for PTMC, whereas dimethyl sulfoxide (DMSO), N-Methyl-2-pyrrolidone (NMP), dimethylformamide (DMF) and propylene carbonate (PC) are solvents for PTMC.

	Non-Solvents		Solvents				
	Water	EtOH	DMSO	NMP	DMF	PC	CHCl_3_
CAS RN	7732-18-5	64-17-5	67-68-5	872-50-4	68-12-2	108-32-7	67-663
MW (g/mol)	18.02	46.069	78.13	99.13	73.1	102.9	119.37
Hildebrand factor (δ SI)	48	26.2	29.7	23.1	24.7	27.2	18.7
Water solubility (%)	100	100	100	100	100	8.3	0.82
Polarity index	10.2	5.2	7.2	6.7	6.4	6.1	4.1
Freezing point (°C)	0	−114.1	18.5	−24.4	−60.4	−55.0	−63.55
Boiling point (°C)	100	78.32	189	202	153	242	61.15
Surface tension (mN/m)	74.9 (5 °C) 72.8 (20 °C) 66.2 (60 °C)	22.4 (20 °C)	43.5 (20 °C)	40.8 (20 °C)	36.8 (20 °C)	41.9 (20 °C)	27.1 (20 °C)

**Table 2:** Characteristics of PTMC-dMA scaffolds. These scaffolds are prepared at a 25% *w*/*w* concentration on ultrapure water at RT. All numbers represent the mean ± SD; ns: *p* > 0.05, * *p* ≤ 0.05, ** *p* ≤ 0.01, *** *p* ≤ 0.001 (one-way ANOVA test with a Tukey’s multiple comparison post-test).

Scaffold Characteristic	PTMC-dMA ^1^	Sc1 (10 min)	Sc2 (60 min)	Sc3 (180 min)	Significance
Gel content (%)		93 ± 5	91 ± 5	86 ± 3	ns
Porosity (%)	-	70 ± 1	75 ± 3	81 ± 2	Sc1-Sc2 **, Sc1-Sc3 ***, Sc2-Sc3 **
Pores/0.86 mm^2^—air side	-	784	129	111	-
Pore diameter—air side (µm)	-	21 ± 18	44 ± 3	52 ± 4	Sc1-Sc2, Sc3 ***, Sc2-Sc3 *
Pores/0.86 mm^2^–water side	-	75	32	5	-
Pore diameter–water side (µm)	-	74 ± 32	98 ± 58	295 ± 50	Sc1-Sc2 *, Sc1-Sc3 ***, Sc2-Sc3 ***
Permeance (L/m^2^ × h × bar)	-	3 ± 3	249 ± 3	267 ± 53	Sc1-Sc2 **, Sc1-Sc3 ***
Young’s modulus (kPa)	2531 ± 338	575 ± 40	617 ± 55	544 ± 54	ns
dL at break (%)	1969 ± 619	122 ± 31	123 ± 35	83 ± 49	ns
Max. stress (kPa)	2312 ± 820	399 ± 61	487 ± 40	275 ± 109	ns
Toughness (N/mm^2^)	2194 ± 1036	4 ± 2	4 ± 3	15 ± 12	ns

^1^ Non-porous PTMC membrane from previous studies used as material control. Adapted with permission from Ref. [14]. Copyright 2020 Iris Allijn.

## Data Availability

Data is contained within the article.

## Supplementary Materials

The following supporting information can be downloaded at: https://www.mdpi.com/article/10.3390/membranes12050453/s1, Figure S1: Polymerization and functionalization; Figure S2: Physical appearance of PTMC membrane-based scaffolds; Figure S3: Time on water determines pore size; Figure S4: Pore size gradient; Figure S5. LX2 cells are viable on the membrane-based scaffolds; Table S1: Membrane-based scaffold defining parameters; Video S1: Preparation of a large Sc1 scaffold; Video S2: Shape memory of an Sc1 scaffold.
