# Supplementary material for: One-Step Fabrication of Porous Membrane-Based Scaffolds by Air-Water Interfacial Phase Separation: Opportunities for Engineered Tissues
Source: Membranes (Basel). 2022 Apr 23;12(5):453. doi: 10.3390/membranes12050453 (PMC9145851; doi:10.3390/membranes12050453)
Supplement: Supplementary file 1 [file membranes-12-00453-s001.zip › membranes-1682520-supplementary.pdf]

Supporting information

One-step fabrication of porous membrane-based scaffolds by air-water interfacial phase separation: opportunities for engineered tissues

Iris Eva Allijn[a*], Nikola Marguerite du Preez[a], Małgorzata Marta Tasior[a], Ruchi Bansal[b], Dimitrios Stamatialis[a]

Iris Allijn [1*], Nikola du Preez [1], Małgorzata Tasior [1], Ruchi Bansal [2] and Dimitrios Stamatialis [1]

[1] Advanced Organ bioengineering and Therapeutics, Faculty of Science and Technology, TechMed Center, University of Twente, 7500 AE Enschede; d.stamatialis@utwente.nl

[2] Translational Liver Research, Department of Medical Cell Biophysics, Faculty of Science and Technology, TechMed Center, University of Twente, 7500 AE Enschede; r.bansal@utwente.nl

* Correspondence: i.e.allijn@utwente.nl

Methods

Human hepatic stellate (LX2) cell culture on PTMC-dMA scaffolds

The cells were seeded on both the air side and water side of the scaffolds at a 100,000 cells/mL concentration. After 24 h, the scaffolds with cells were washed twice with PBS, fixed with ice-cold acetone:methanol (1:1) for 30 minutes. After fixation, the fixation solution was decanted and air-dried. The scaffolds were rehydrated with PBS and mounted using DAPI mounting medium. The brightfield and fluorescent (in DAPI channel) images were made using inverted microscope (Nikon).

Figures

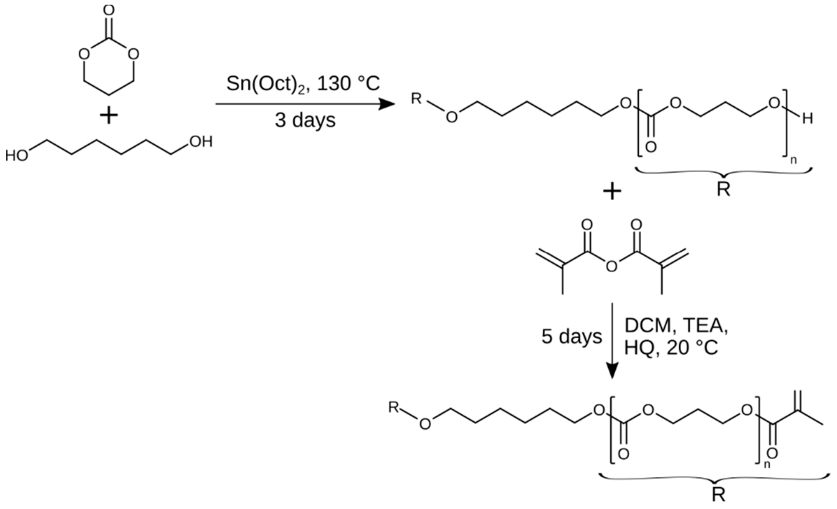

Figure S1. Polymerization and functionalization. Ring opening polymerization of trimethylene carbonate with hexanediol and subsequent functionalization of the hydroxyl groups with methacrylic anhydride. TEA = triethylamine, DCM = dichloromethane, HQ = hydroquinone.

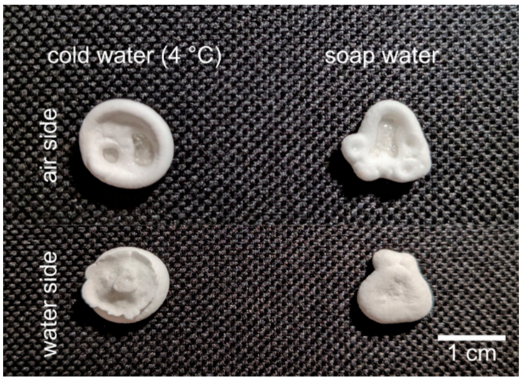

Figure S2. **Physical appearance of PTMC membrane-based scaffolds.** When the polymer solution is cast on cold water or soap water, no spreading occurs and subsequently sinks to the bottom.

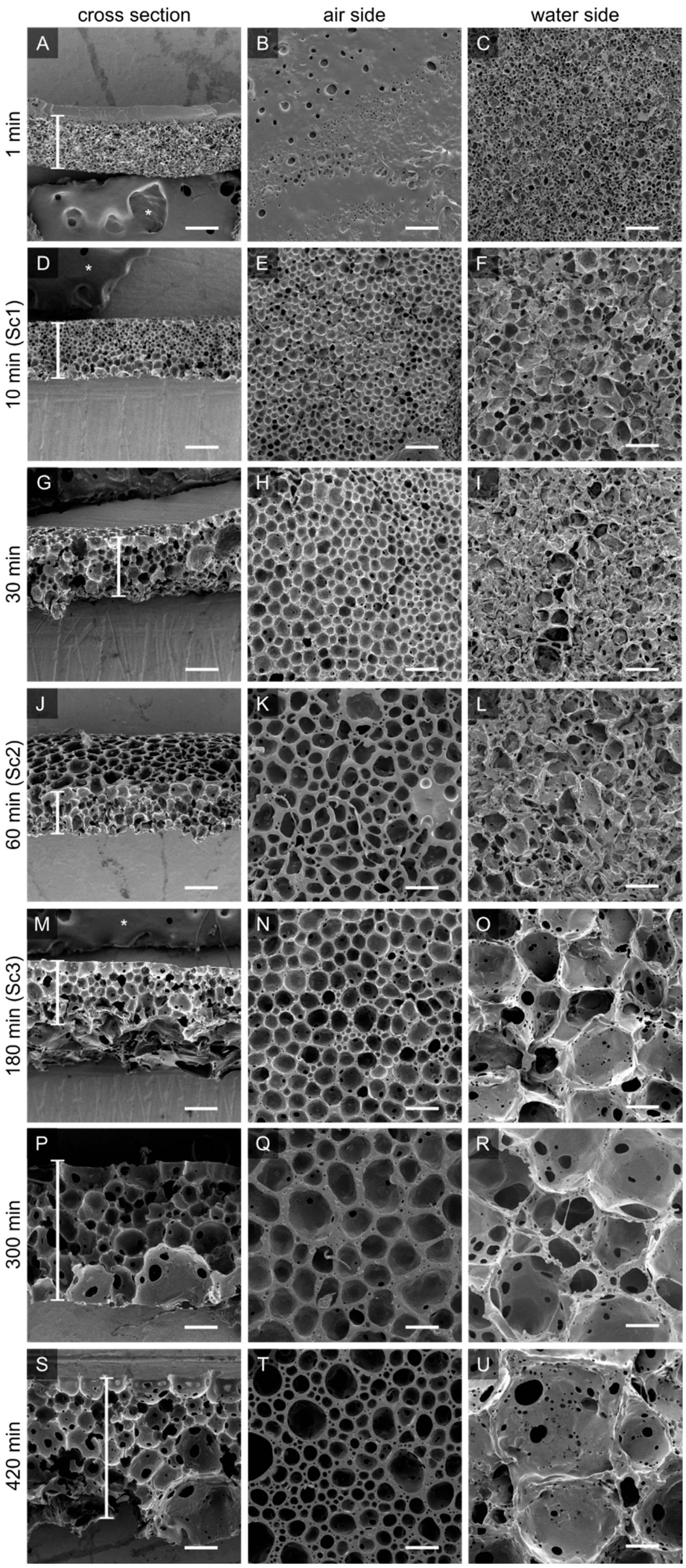

cross section
air side
water side
1 min
10 min (Sc1)
30 min
60 min (Sc2)
180 min (Sc3)
300 min
420 min
A
B
C
D
E
F
G
H
I
J
K
L
M
N
O
P
Q
R
S
T
U

Figure S3. Time on water determines pore size. Scanning electron micrographs are shown for PTMC scaffolds with an initial concentration of 25% (w/w) prepared by floating on water for 1 min (A-C), 10 min (Sc1, D-F), 30 min (G-I), 60 min (Sc2, J-L), 180 min (Sc3, M-O), 300 min (P-R) and 420 min (S-U). Scaffold pore sizes increase with increased time on water before photo-crosslinking. Scaffold thickness is indicated by a white vertical bar. Scale bars are 200 μm. The asterisk (*) indicates the carbon tape which was used to stick the samples to the SEM.

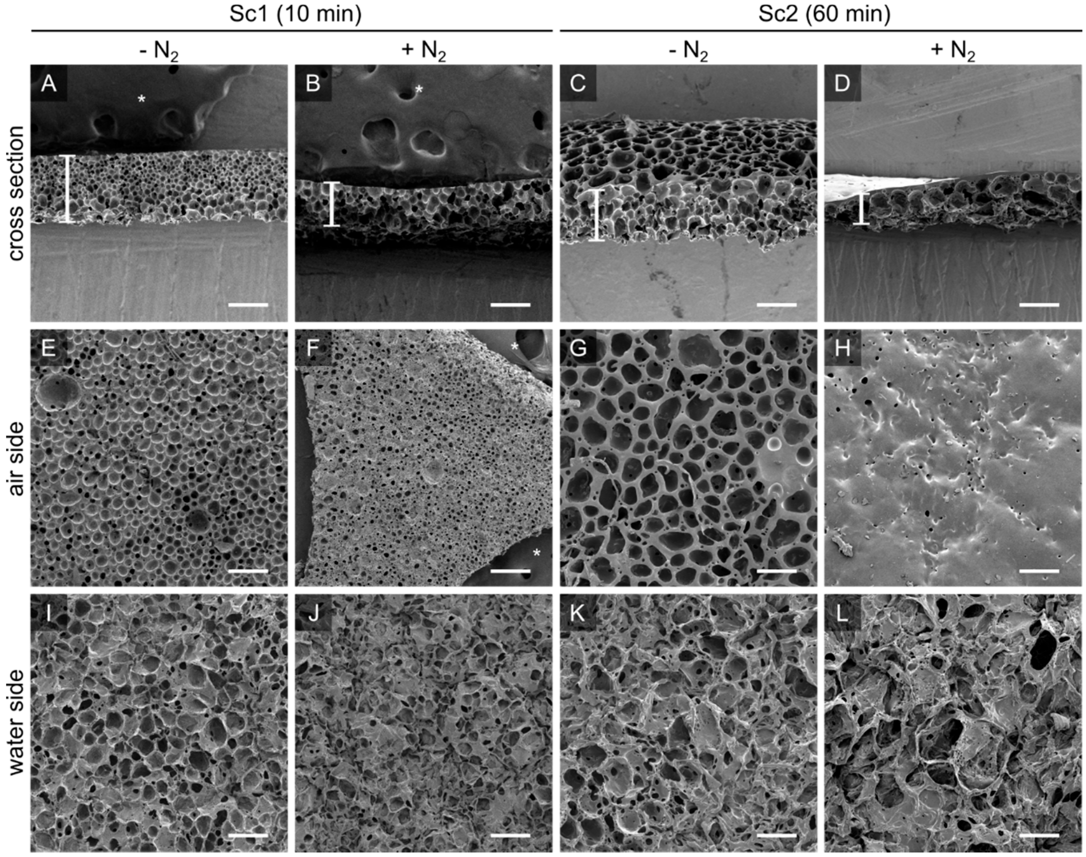

Figure S4. Pore size gradient. The pore size gradient can be enhanced by applying a N_2 top flow before photo-crosslinking. Scanning electron micrographs are shown for PTMC scaffolds Sc1 and Sc2 with an initial concentration of 25% (w/w) prepared by floating on water for 10 min and 60 min. A clear decrease in air surface pore sizes can be observed after applying the N_2 top flow (panels F and H). The top panels (A-D) show the cross sections of the scaffolds, with the air side facing upwards. Scaffold thickness is indicated by a white vertical bar. The middle panels (E-H) show the air side and the bottom panels (I-L) show the water side of the scaffolds. Scale bars are 200 μm. The asterisk (*) indicates the carbon tape which was used to stick the samples to the SEM stub.

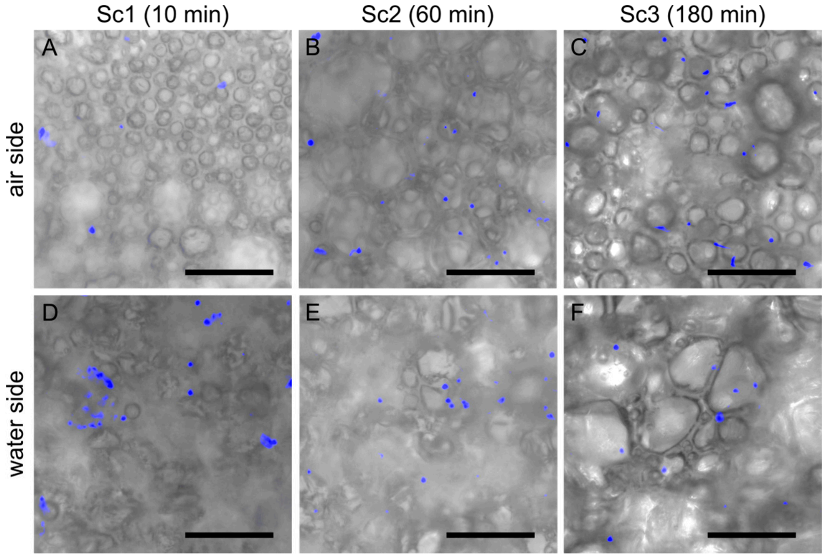

Figure S5. LX2 cells are viable on the membrane-based scaffolds. Bright field imaged are shown with a Dapi (blue) overlay of LX2 cell nuclei on PTMC-dMA scaffolds Sc1 (A+D), Sc2 (B+E) and Sc3 (C+F). Scale bars are 200 µm.

Tables

Table S1. Membrane-based scaffold defining parameters. Selected parameters are depicted in bold.

Parameter	General description	Air side	Water side
Polymer concentration: PTMC-dMA in DMSO			
15% w/w	uneven morphology	no pores	no pores
20% w/w	dense inner layer	irregular pores	small pores
**25% w/w**	even pore distribution	smooth, round pores	rough, honeycomb-like pores
30% w/w	too viscous to handle and spread on the water	-	-
Solvent: 25% w/w PTMC-dMA			
**DMSO**	even pore distribution	smooth, round pores	rough, honeycomb-like pores
NMP	irregular with large pores	irregular pores	smooth, some deep pores
DMF	thin, one layer of pores	smooth, few small pores	smooth, indentations
PC	no scaffold formation	-	-
$CHCl_3$	very thin, fragile	no pores	no pores
Water temperature: 25% w/w PTMC-dMA in DMSO			
4 ˚C	sinks to the bottom	-	-
**20 ˚C**	even pore distribution	smooth, round pores	rough, honeycomb-like pores
40 ˚C	fast spreading, tears on the edges	round pores	rough, honeycomb-like pores
Time on water: 25% w/w PTMC-dMA in DMSO at 20 ˚C			
1 min	only porous on water side	no pores	rough, honeycomb-like pores
**10 min (Sc1)**	even pore distribution	smooth, round pores	rough, honeycomb-like pores
30 min	some large pores inside the scaffold	round large pores with small pores	rough, some pores
**60 min (Sc2)**	even pore distribution	larger pores with thick walls	rough, honeycomb-like pores
**180 min (Sc3)**	even pore distribution	pores sizes as 60 min with small pores in pore walls	large, rough honeycomb-like pores
300 min	even pore distribution with pore size gradient	round pores, larger than 60 min with thick walls	large, rough, open honeycomb-like pores with smaller pores inside
420 min	uneven pore distribution	round, deep pores, larger than 300 min with thick walls	very large, rough honeycomb-like closed pores with smaller pores inside

Videos

Video S1. Preparation of a large Sc1 scaffold. PTMC-dMA (25% w/w) in DMSO is dropwise added to milliQ water in a petridish (Ø = 9 cm) and left for 10 minutes to form. Afterwards the scaffold is UV-cured and solvents are extracted (see also Fig. 1).

Video S2. Shape memory of an Sc1 scaffold. The dried scaffold can be stretched, rubbed and folded without losing its shape.
